# Supplementary material for: Macro- and atomic-scale observations of a one-dimensional heterojunction in a nickel and palladium nanowire complex
Source: Nat Commun. 2022 Mar 4;13:1188. doi: 10.1038/s41467-022-28875-8 (PMC8897505; doi:10.1038/s41467-022-28875-8)
Supplement: Supplementary file 1 — Supplementary Information [file 41467_2022_28875_MOESM1_ESM.pdf]

Supplementary information for

Macro- and atomic-scale observations of a one-dimensional heterojunction in a nickel and palladium nanowire complex

Masanori Wakizaka,[1]* Shohei Kumagai,[1] Hashen Wu,[1] Takuya Sonobe,[1] Hiroaki Iguchi,[1] Takefumi Yoshida,[1] Masahiro Yamashita,[1,2]* and Shinya Takaishi[1]*

[1]Department of Chemistry, Graduate School of Science, Tohoku University, 6-3 Aramaki-Aza-Aoba, Aoba-Ku, Sendai 980-8578, Japan. Fax: +81-22-795-6548; Tel: +81-22-795-6545.
[2]School of Materials Science and Engineering, Nankai University, Tianjin 300350, P. R. China

E-mail: shinya.takaishi.d8@tohoku.ac.jp, yamasita@agnus.chem.tohoku.ac.jp, masanori.wakizaka.a7@tohoku.ac.jp

Contents

**Supplementary Table 1**	Cell parameters for **1** and **2**
**Supplementary Fig. 1**	Optical and SEM images for the **1–2** heterostructure
**Supplementary Fig. 2**	SEM-EDS spectra for the **1–2** heterostructure
**Supplementary Fig. 3**	Polarized reflectivity spectra of the **1** and **2** region in the **1–2** heterostructure
**Supplementary Fig. 4**	Polarized reflectivity spectra with IR region.

Supplementary Table 1 | Cell parameters for **1** and **2**

Axis	**1** (Å)[21]	**2** (Å)[21]	Difference (%)
a	23.587(8)	23.884(5)	–1.2
b	5.161(2)	5.296(1)	–2.5
c	7.121(1)	7.067(4)	0.8

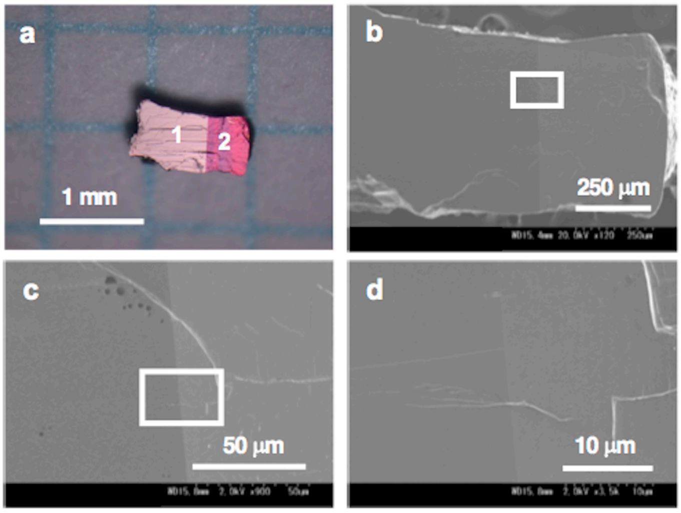

Supplementary Fig. 1 | (**a**) Optical and (**b**) SEM images for the **1–2** heterostructure. The SEM images of (**c**) and (**d**) shows magnification in the area of white square, respectively. Scale bars, 1 mm, 250 µm, 50 µm and 10 µm (for **a**, **b**, **c**, and **d**, respectively). Source data are provided as a Source Data file.

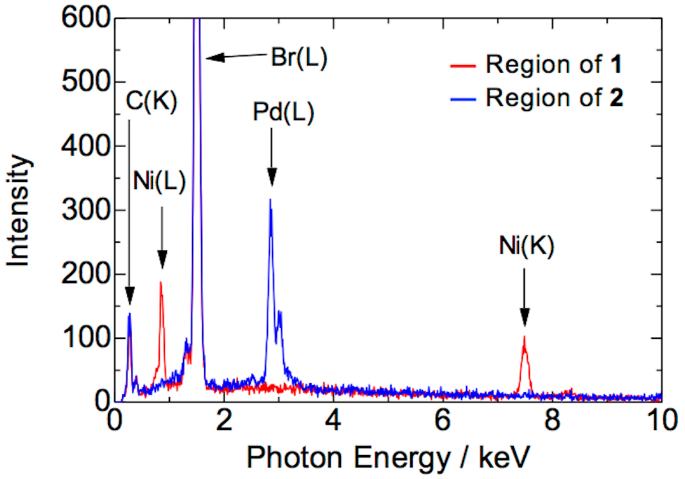

Supplementary Fig. 2 | SEM-EDS spectra for the **1–2** heterostructure. Source data are provided as a Source Data file.

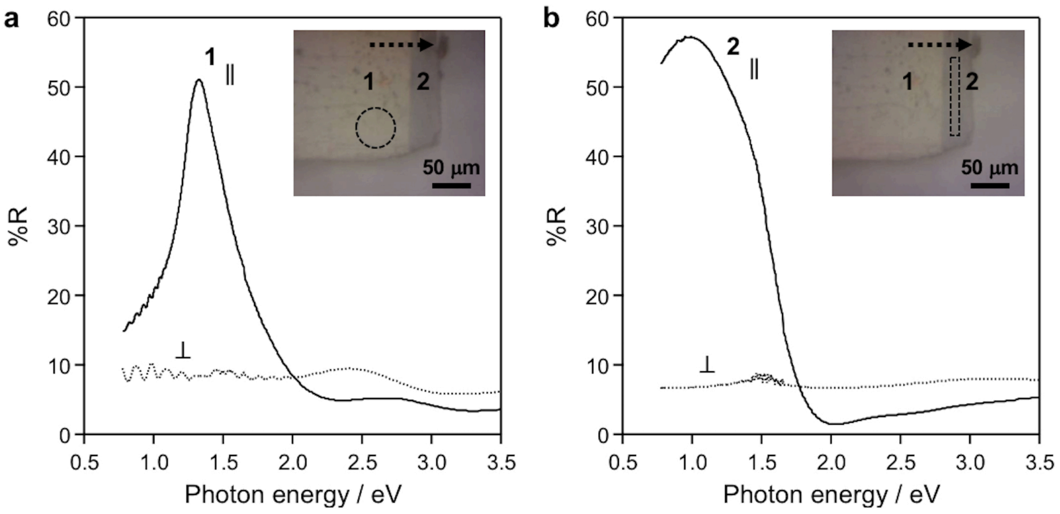

Supplementary Fig. 3 | The polarized reflectivity spectra for parallel (∥: black solid line) and perpendicular (⊥: black dotted line) toward the chain direction of the (**a**) **1** and (**b**) **2** region in the **1–2** heterostructure. The insets show optical images with apertures (ϕ = 50 μm and 10 μm × 100 μm). Scale bars 50 μm (for **a** and **b**). Source data are provided as a Source Data file.

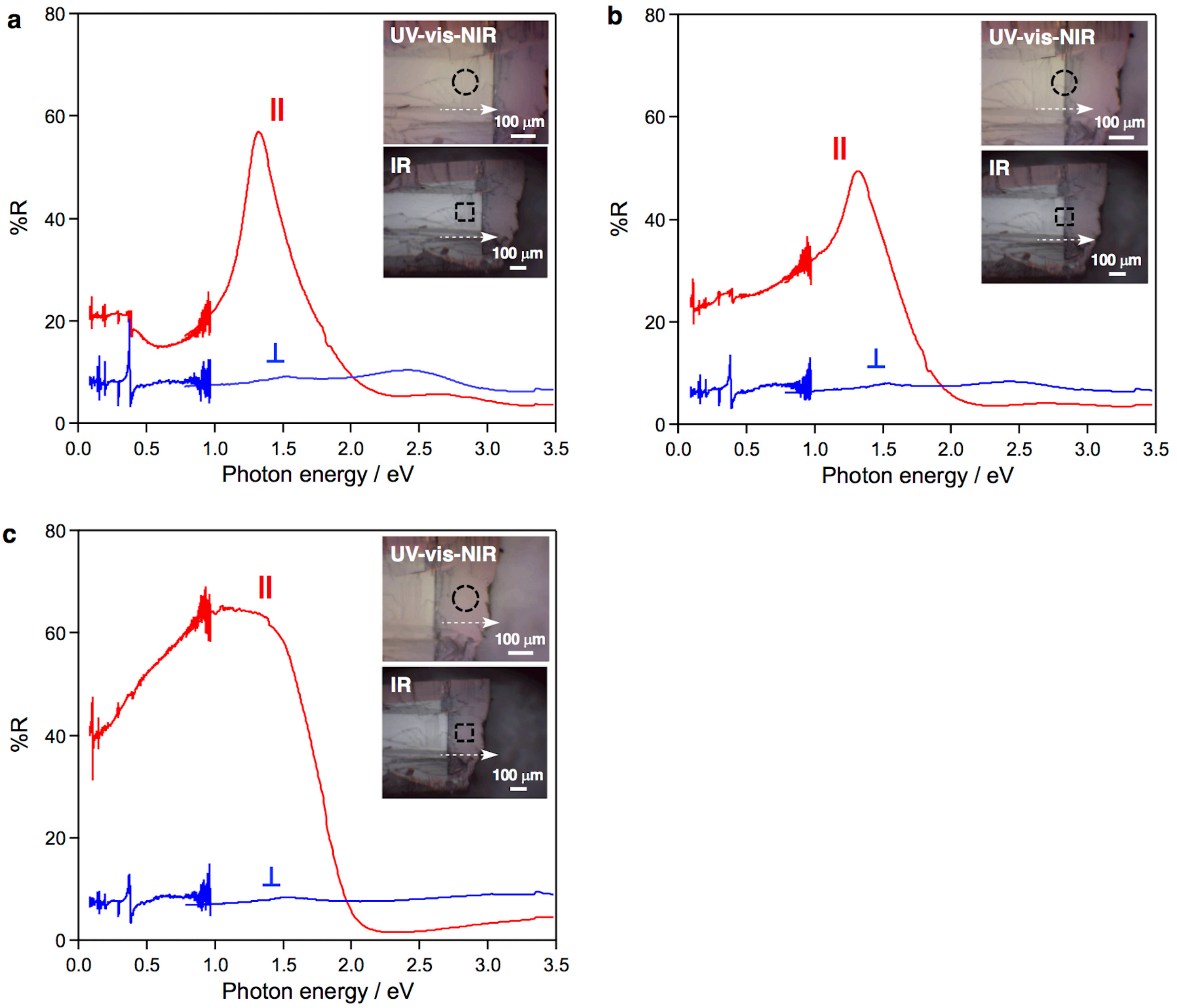

Supplementary Fig. 4 | The polarized reflectivity spectra for parallel (∥: red lines) and perpendicular (⊥: blue lines) toward the chain direction of the (**a**) **1**, (**b**) heterojunction, and (**c**) **2** regions in the **1**–**2** heterostructure. The insets show optical images with apertures (UV-vis-NIR region: $\phi = 100$ μm; IR region: 100 μm × 100 μm). Scale bars 100 μm (for **a**–**c**). Source data are provided as a Source Data file.
